# Evaluation of *Drimys winteri* (Canelo) Essential Oil as Insecticide against *Acanthoscelides obtectus* (Coleoptera: Bruchidae) and *Aegorhinus superciliosus* (Coleoptera: Curculionidae)

**DOI:** 10.3390/insects11060335

**Published:** 2020-05-30

**Authors:** Jocelyne Tampe, Javier Espinoza, Manuel Chacón-Fuentes, Andrés Quiroz, Mónica Rubilar

**Affiliations:** 1 Technology and Processes Laboratory, Department of Chemical Engineering, Faculty of Engineering and Science, Universidad de La Frontera, Avenida Francisco Salazar 01145, Temuco 4811230, Chile; 2 Scientific and Technological Bioresource Nucleus, BIOREN, Universidad de La Frontera, Avenida Francisco Salazar 01145, Temuco 4811230, Chile; 3 Laboratorio de Química Ecológica, Departamento de Ciencias Químicas y Recursos Naturales, Universidad de La Frontera, Casilla 54-D, Avenida Francisco Salazar 01145, Temuco 4811230, Chile; javier.espinozama@usach.cl (J.E.); manuel.chacon@ufrontera.cl (M.C.-F.); quirozandre@gmail.com (A.Q.); 4 Centro de Excelencia en Investigación Biotecnológica Aplicada al Medio Ambiente (CIBAMA), Facultad de Ingeniería y Ciencias, Universidad de La Frontera, Avenida Francisco Salazar 01145, Temuco 4811230, Chile

**Keywords:** bean weevil, raspberry weevil, toxic effect, repellent effect

## Abstract

Adverse effects caused by synthetic pesticides have increased interest in plant-derived insecticidal compounds, in particular essential oils, as a more compatible and ecofriendly alternative for pest control of economic importance. For this reason, the essential oil isolated from leaves and shoots of *Drimys winteri* (J.R. Forster & G. Forster)—also named canelo (CEO)—was investigated for its chemical profile and insecticidal action against *Acanthoscelides obtectus* (Say)—one of the most important post-harvest pests of dry beans in the world—and *Aegorhinus superciliosus* (Guérin)—a significant pest of fruit trees in Chile. The analysis by gas chromatography, paired with mass spectrometry (GC/MS) determined 56 compounds, corresponding to 92.28% of the detected compounds. Elemol (13.54%), *γ*-eudesmol (11.42%), *β*-eudesmol (8.49%), *α*-eudesmol (6.39%), *α*-pinene (7.92%) and *β*-pinene (5.17%) were the most abundant. Regarding the bioactivity of the CEO, the results demonstrated toxicological effects against *A. obtectus*. A concentration of 158.3 µL L^−1^ had a mortality rate of 94% after 24 h exposure. The LC_50_ and LC_90_ values at 24 h were 60.1 and 163.0 µL L^−1^. Moreover, behavioral bioassays showed a repellent effect against *A. superciliosus* with a dose of one microliter of CEO. Both sexes of the raspberry weevil stayed for very short times in the treated area with the oil (<0.8 min), showing a homogeneous repellency in the species. The overall data suggest that canelo leaves and shoots essential oil has an insecticide effect and is worth exploring to better understand the synergistic relationship between the compounds present in the essential oil.

## 1. Introduction

Currently, the study and use of essential oils (EOs) is an interesting and potential tool to develop botanical insecticides safer for health and the environment [1,2]. They are biosynthesized in aromatic plants as secondary metabolites and they play a protective role against biotic factors, such as bacteria, fungi, viruses and insects [1,2,3,4]. EOs are composed of a wide variety of volatile compounds, mainly terpenes at high concentrations (between 20%–70%) in addition to other chemical groups present in trace amounts [1]. In overall, the bioactivity of EOs has been recorded to act by contact, ingestion and as fumigants as well as by their antifeedant or repellent action on different pest insects [5,6,7]. In this context, recent investigations have showed the insecticidal activity of different EOs to manage *Prostephanus truncatus* (Horn) (Coleoptera: Bostrychidae) and *Trogoderma granarium* (Everts) (Coleoptera: Dermestidae) infestations [8] against different flies; *Calliphora vomitoria* (L.) (Diptera: Calliphoridae) [9], *Ceratitis capitata* (Wiedemann) and *Anastrepha fraterculus* (Wiedemann) (Diptera: Tephritidae) [10] against *Callosobruchus maculatus* (Fabricius) (Coleoptera: Chrysomelidae), the main cowpea pest [11] and against two lepidopteran pests, *Spodoptera frugiperda* (Walker) and *Anticarsia gemmatalis* (Hübner) (Lepidoptera: Noctuidae) [12], proving to be a useful and effective tool in reducing harmful insect populations and the use of conventional pesticides [13].

*Drimys winteri* (J.R. Forster & G. Forster) (Winteraceae)—commonly known as canelo, voigue or boighe—is a perennial tree growing in the sub-Antarctic forests of Chile and Argentina [14]. This species is characterized as having great ecological plasticity [15], growing in wetlands and marshes [16] and even in lands left bare due to fire [17]. It is described as a tree with a thick and soft bark, reaching up to 30 m height, with large, ovate very aromatic and smooth leaves [18]. In Chilean indigenous culture, canelo is known as a medicinal plant [18,19], and has been widely used by the Mapuche people as a sacred tree, as it symbolizes the Axis Mundi [20], in addition to its healing, disinfectant and antibacterial properties [21,22]. Canelo bioactivity has been reported from its compounds and EOs obtained from its stems, leaves and bark. Phytochemical studies have shown the activity of polygodial and drimenol sesquiterpenes for medical applications [23], as well as pest control [24,25]. Moreover, the *D. winteri* EO has produced insecticidal activity against aphids [26], stored grain insects [27] and weevils [28], showing great potential as a natural pesticide. However, there are few the studies that report the insecticidal potential of *D. winteri* EO. Therefore, our work seeks to expand the knowledge of canelo insecticidal bioactivity towards two important pests of the order Coleoptera—the bean weevil *A. obtectus*, and the raspberry weevil *A. superciliosus*.

The bean weevil, *Acanthoscelides obtectus* (Say) (Coleoptera: Bruchidae), is one of the world’s most important post-harvest pests in dry beans, *Phaseolus vulgaris* (L.) (Fabaceae) [29,30]. It is mainly found in South America, Africa and the Mediterranean [31,32,33], where the adult attacks bean seeds while they are still in the field and continues to cause damage during storage. This can cause the total loss of stored bean seeds [34,35] as the larvae enter the bean seeds to feed and develop from larva to adult inside the seeds [36]. Some researchers have reported losses around 7%–40% of stored bean seeds [31,37], which equates to a loss of 1.59–9.12 million tons annually in the world caused by this bruchid [38]. On the other hand, the raspberry weevil, *Aegorhinus superciliosus* (Guérin) (Coleoptera: Curculionidae), is a significant pest of fruit trees such as European hazelnut (*Corylus avellana* L.) (Betulaceae), blueberry (*Vaccinium corymbosum* L.) (Ericaceae), raspberry (*Rubus idaeus* L.) (Rosaceae) and other minor fruit trees. The larvae of this weevil attack the plant’s root system, boring into the main root, affecting water and nutrient uptake and causing the death of the plant. Moreover, adult weevils feed on the leaves and shoots of the season, affecting vegetative growth [39,40]. Currently, broad-spectrum synthetic pesticides such as pyrethroids and organophosphates are the most used to control these pests [41,42]. However, their application on stored dry beans and fruit orchards has increased public concern over pesticide safety and environmental damage [43,44,45,46]. In this respect, our study’s aim is to promote research with endemic plant species in Chile as a natural alternative more compatible with safer pest control approaches. Hence, we assessed the insecticidal potential of canelo essential oil as a toxicological agent against *A. obtectus*, a stored dried bean pest and as a repellent against *A. superciliosus*, a fruit tree pest, and we determined the *D. winteri* EO chemical profile by gas chromatography coupled to mass spectrometry (GC-MS).

## 2. Materials and Methods

### 2.1. Plant Material and Essential Oil Extraction

*Drimys winteri* aerial parts were collected in the fall of 2017 from Vilcún (38°40’08.1” S, 72°16’22.9” W), La Araucanía, Chile. Its identity was confirmed by comparing macroscopic and microscopic morphologic characteristics to Chilean flora and specimens in the herbarium at the Universidad de Concepción, Chile. Fresh leaves and shoots were washed with distilled water to remove any residue. The extraction of the essential oil from leaves and shoots of canelo—hereafter referred to as CEO—was performed according to Zapata and Smagghe [27]. Briefly, chopped leaves and shoots of *D. winteri* (0.4 kg) were subjected to hydrodistillation for 4 h in a Clevenger apparatus. Then, the CEO was dried over anhydrous sodium sulfate [47,48].

### 2.2. Essential Oil Analysis

The CEO was analyzed with gas chromatography coupled to mass spectrometer (GC/MS), using the following instrumentation: a Thermo Focus GC (Thermo Fisher Scientific, Waltham, MA, USA) coupled to a Thermo DSQ quadrupole mass spectrometric detector with an integrated data system (Xcalibur 2.0, Thermo Fisher Scientific, Inc., Waltham, MA, USA). One microliter aliquot of the CEO diluted in hexane was injected at a concentration of 1 μg μL^−1^ in a capillary column BPX5 (30-m length, ×0.25-µm film thickness, 0.25-mm inner diameter, SGE Forte, Trajan Scientific and Medical, Ringwood, Victoria, Australia) in splitless mode. The operating conditions were on-column injection: injector temperature of 250 °C, transfer line temperature of 250 °C; detector temperature of 250 °C; carrier gas: He at 1.0 mL min^−1^, oven temperature program: 40 °C for 2 min, increased to 250 °C at 5 °C/min, followed by 250 °C for 5 min. The mass spectra were obtained at an ionization voltage of 70 eV. Recording conditions employed a scan time of 1.5 s and a mass range of 30–400 amu. The compounds were identified based on the comparisons of the mass spectra with a library database (NIST ver. 2.0, NIST, Gaithersburg, MD, USA) [49], and by comparisons of their retention indices with those reported in the literature for the same column type [50]. The sample was analyzed once.

### 2.3. Insects

*Acanthoscelides obtectus*: The original population of *A. obtectus* was collected from contaminated stored bean seeds in La Araucanía Region, Chile in 2017. The population started with at least 300 individuals that were reared on a white bean variety purchased from the local market and previously maintained in a freezer at −20 °C. Adult couples were maintained in 1.2-L glass jars covered with fabric. Females oviposited on beans and the larvae developed inside the beans until adult emergence. The laboratory rearing conditions were 25 °C, 65% relative humidity and a photoperiod of 14 h light, 10 h dark [51].

*Aegorhinus superciliosus*: Adult insects were collected manually from a blueberry (*V. corymbosum*) plantation in Collipulli (38°00’44.8” S, 72°08’32.4” O), La Araucanía Region, Chile during the 2017–2018 summer season. Then, they were transferred to the Chemical Ecology Laboratory at the Universidad de La Frontera for their acclimatization. The insects were maintained under a 16:8 light-dark cycle at 20 °C on fresh blueberry leaves and shoots [48]. Weevils were allowed free access to water. Before the assays (24 h), each insect was kept in an individual petri dish and starved until the assays [52].

### 2.4. Toxicological Bioassays for Acanthoscelides obtectus

**Figure 1:**
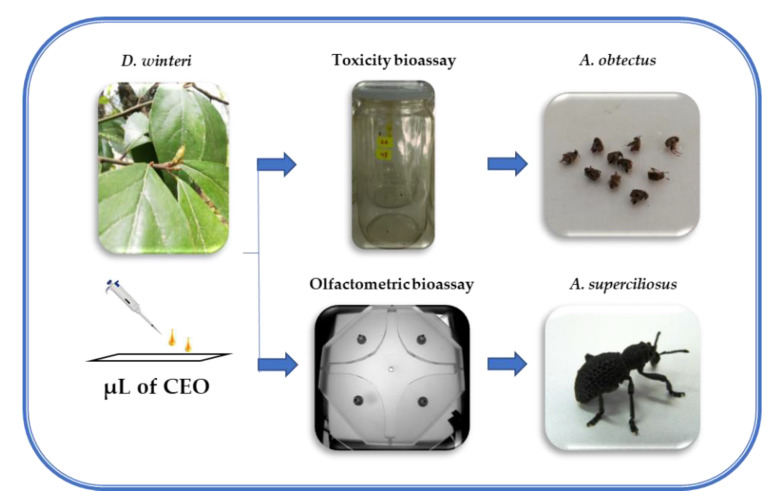
Bioassay scheme for each insect.

The inhalation bioassay against *A. obtectus* was performed according to methodology reported by Ayvaz et al. [51] with modifications. Briefly, ten unsexed 1–2-day-old adult bruchids (F_1_) were placed in a glass jar (1.2 L) fitted with a screw lid with a filter paper strip (3 × 3 cm) attached to the center of the internal face of the lid (Figure 1). Different doses of pure CEO (10, 70, 130 and 190 µL) were applied to the strip in concentrations of 8.3, 58.3, 108.3 and 158.3 µL L^−1^, respectively. The control jars did not have CEO on the strip. For evaluating the mortality of bruchids due to CEO, ten insects were placed in a jar (the jars were then tightly sealed and kept at 25 ± 2 °C under a 14:10 light-dark cycle and 65% humidity) and replicated nine times for each concentration. All insects were assessed in each group by stimulating each bruchid with a brush. Insects that did not respond were considered dead. All insects at the end of their respective evaluation time were discarded. Then, mortality values were used to calculate the lethal concentration for the death of 50% (LC_50_) and 90% (LC_90_) of the individuals caused by the CEO.

### 2.5. Repellency Bioassays for Aegorhinus superciliosus

The behavioral response of both sexes of *A. superciliosus* produced by the CEO was tested using a four-arm olfactometer described by Parra et al. [52]. This assay was performed according to the methodology described by Tapia et al. [53], based on the residence time that each insect remained in each arm of the olfactometer. The olfactometer areas are divided into five zones: two arms enriched with the EO volatiles (stimuli), two arms as control zones and one central square zone (decision zone) connected to air flow (800 mL min^−1^) generated for carrying the volatile stimuli into the olfactometer (Figure 1). The dose used was according to Espinoza et al. [47], where 1 µL of pure CEO was applied on Whatman N° 1 filter paper (0.5-cm-wide by 3.5-cm-long) and placed in glass tubes (7-cm-long; 1.5-cm inner diameter) in two different arms of the olfactometer. The time that each *A. superciliosus* spent in each arm of the olfactometer was recorded for 10 min and replicated 20 times per sex [48]. A new individual was used in each replicate of the experiments and then discarded. After each replicate the olfactometer was cleaned with ethanol [52].

### 2.6. Data Analysis

The statistics software Statistix 10 (2014, Tallahassee, FL, USA) was used to analyze the data. For comparing mortality between time (24, 48 and 72 h) and concentrations (control, 8.3, 58.3, 108.3 and 158.3 µL L^−1^) on the *A. obtectus*, a one-way analysis of variance (ANOVA) was carried out followed by a Tukey’s test at 95% confidence. These data were expressed as a percentage for each treatment and an arcsine square root transformation was carried out to meet the assumption of homogeneity of variance and a normal distribution. To determine lethal doses (LC_50_ and LC_90_) for each time, a Probit analysis was performed with a logit distribution in response to the binary dependent variable (alive or dead) [54]. Values of *p* ≤ 0.05 were considered significant. Results are expressed as means and their corresponding standard error. Moreover, the data obtained from the olfactometric bioassays were expressed as the average of the time spent in each arm of the olfactometer (min) ± SE and were analyzed by the nonparametric Friedman test (*p* ≤ 0.05) followed by the Conover test [55]. Furthermore, a Wilcoxon test was performed to analyze the sex effects on the insect’s olfactometric behavior.

## 3. Results

### 3.1. Yield and Chemical Profile of Drimys winteri Essential Oil

**Figure 2:**
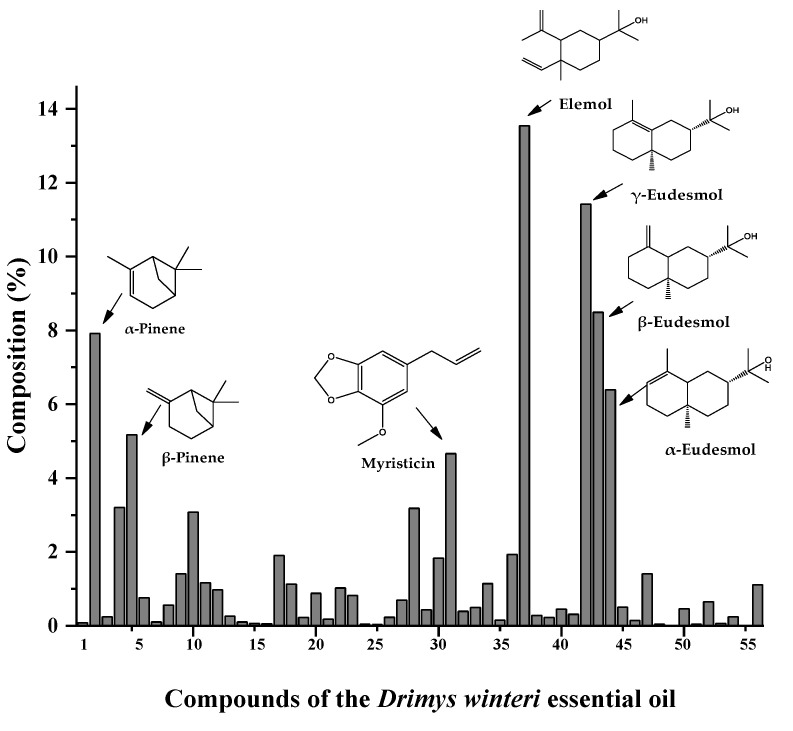
Chemical profile of *D. winteri* leaves and shoots essential oil and main compounds. For the key to identifying peaks, see Table 1.

The crushed fresh leaves and shoots (0.4 kg) of *D. winteri* subjected to hydro-distillation gave a pale yellow EO with a yield of 0.21% (*w/w*). The CEO was analyzed by GC/MS (Table 1) and 56 compounds were identified corresponding to 92.2% of all detected compounds. Seventeen monoterpenes (28.0%), 13 hydrocarbonated (23.5%) and 4 oxygenated (4.4%), 28 sesquiterpenes (56.6%), 15 hydrocarbonated (11.5%) and 13 oxygenated (45.1%), 3 diterpene hydrocarbons (1.15%), 2 phenylpropanoids (4.8%), 5 ester compounds (0.4%) and one saturated hydrocarbon (1.11%) were present in the CEO. The oxygenated sesquiterpenes elemol (13.5%), *γ*-eudesmol (11.4%), *β*-eudesmol (8.4%) and *α*-eudesmol (6.3%) and the monoterpene hydrocarbons *α*-pinene (7.9%) and *β*-pinene (5.1%) were the most abundant compounds in the CEO (Figure 2).

The oxygenated sesquiterpenes elemol (13.5%), *γ*-eudesmol (11.4%), *β*-eudesmol (8.4%) and *α*-eudesmol (6.3%), and the monoterpene hydrocarbons *α*-pinene (7.9%) and *β*-pinene (5.1%) were the most abundant compounds in the CEO (Figure 2).

### 3.2. Mortality and Toxicity Bioassay for A. obtectus

**Figure 3:**
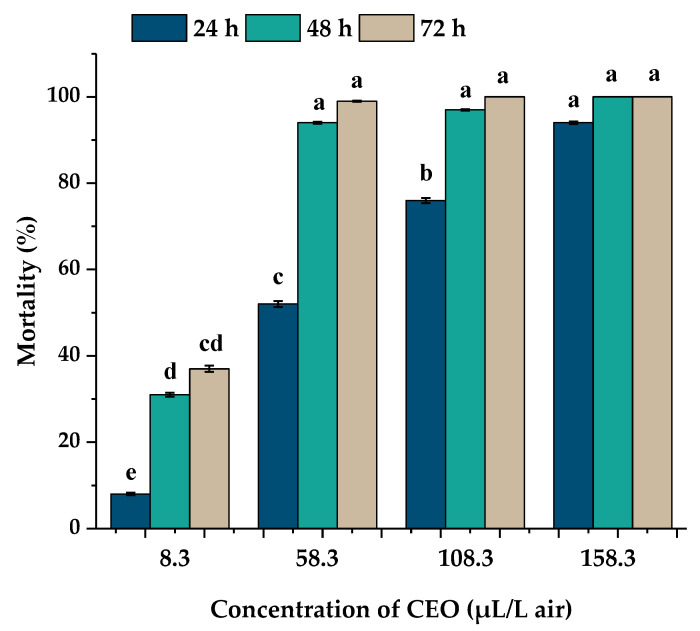
Mortality percentage of the *Acanthoscelides obtectus* adults after time exposure of 24, 48 and 72 h at different concentrations (8.3, 58.3, 108.3 and 158.3 µL L^−1^) of *Drimys winteri* essential oil. Different letters above bars indicate significant differences among doses (*p* < 0.05) according to the ANOVA and Tukey’s tests. Error bars mean standard error. Mortality data (%) were subjected to an arcsine square root transformation for normality assumptions before one-way analysis of variance (ANOVA) and Tukey’s tests.

Results in Figure 3 show that the toxicological activity of the essential oil extracted from *D. winteri* against *A. obtectus* adults was significantly influenced (*F*_120,134_ = 8.4; *p* ≤ 0.001) by the concentration used and, interestingly, the activity also increased when insects were subjected to a longer exposure time. When ascending doses of CEO (8.3, 58.3, 108.3 and 158.3 µL L^−1^) were evaluated, it was observed that the mortality rates increased from 8% to 94% at 24 h of exposure, showing a significant difference among the four evaluated doses (*p* < 0.05). Then, by increasing the exposure time from 24 h to 48 h and 72 h, the lower concentration (8.3 µL L^−1^) achieved a higher mortality from 8% (24 h) to 31% and 36%, respectively, showing a significant difference only between 24 h compared to 48 h and 72 h exposure (*p* < 0.05). Similar results were observed with 58.3 and 108.3 µL L^−1^ of CEO, where there was a significative increase (*p* < 0.05) between the 24 h respect to 48 h and 72 h exposure. The concentration of 58.3 µL L^−1^ caused an increased mortality rate from 54% (24 h) to 94% and 99% at 48 h and 72 h while the concentration 108.3 µL L^−1^ increased the mortality from 75% (24 h) to 97% and 100% at 48 h and 72 h, respectively. In both concentrations, there was no significant difference between 48 h and 72 h (*p* > 0.05). Finally, the higher concentration (158.3 µL L^−1^) achieved statistically similar mortality rates at all exposures times (*p* > 0.05).

After 24 h, median lethal concentration (LC_50_) and lethal concentration (LC_90_) values calculated by Probit analysis were 60.1 and 163.0 µL L^−1^. With the increased exposure time (48 and 72 h) the LC_50_ and LC_90_ values decreased to 14.8 and 40.1 µL L^−1^ and 11.1 and 30.2 µL L^−1^, respectively (Table 2).

### 3.3. Repellency Bioassays for A. superciliosus

**Figure 4:**
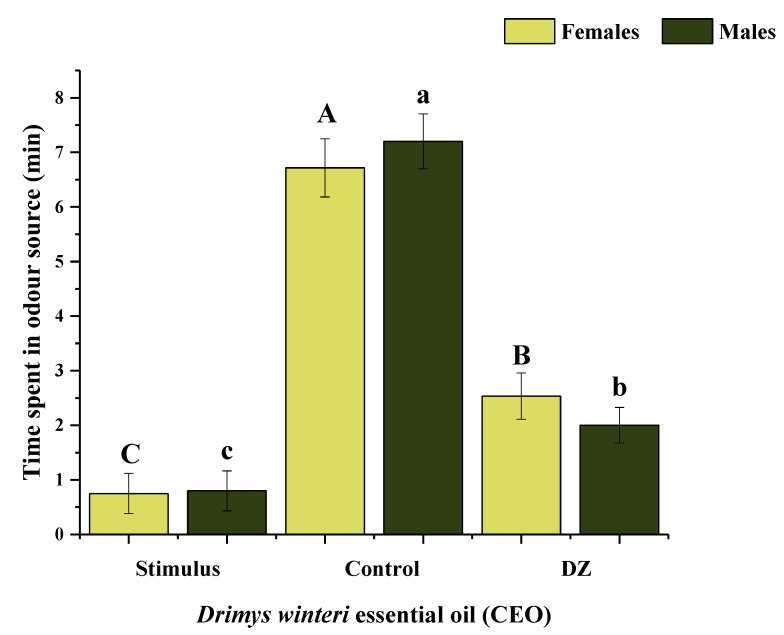
Average time spent (min) (±SE) by both sexes of *Aegorhinus superciliosus* on *Drimys winteri* essential oil (1 µL) in a four-arm olfactometer. Different letters indicate significant differences among zones (stimulus, control and DZ—decision zone) (*p* ≤ 0.05) based on the nonparametric Friedman test followed by the Conover test. *n* = 20 per sex.

The olfactometric response of *A. superciliosus* to CEO indicated that both females and males were repelled by this essential oil (*p* < 0.05). The average time spent by the females in the stimulus source (CEO) was significantly less (0.7 ± 0.4 min) than in the control (6.7 ± 0.5 min; *F* = 24.4; *df* = 2; *p* ≤ 0.001). Similar behavior was observed in the males of the species, where the average spent time in the stimulus was significantly lower (0.8 ± 0.4 min) than the control (7.2 ± 0.5 min; *F* = 39; *df* = 2; *p* ≤ 0.001). In addition, the behavior of the raspberry weevil exhibited a significant response to the decision zone compared to the stimulus and control (*p* < 0.05) (Figure 4). Finally, there were no significant differences in the behavioral response between both sexes of *A. superciliosus*, which indicates a repellent homogeneous effect on the species (*p* > 0.05).

## 4. Discussion

The results indicated that our CEO obtained by hydro-distillation had a yield of 0.21% (*w/w*), which is consistent with what was reported by Verdeguer et al. [56] and Muñoz et al. [57] who used the same plant material and distillation method, with a yield of 0.22% and 0.26%, respectively. Hydro-distillation is the method most used for the extraction of the EO from *D. winteri*, showing a yield variable (0.05%–4.18%) according to amount of vegetal material used or different tissue of extraction (leaves, stem bark and wood) [27,56,57,58,59,60]. Only one work used a different distillation method (Soxhlet-Steam-distilled) with a yield (1.21%) similar to the other studies [61]. Other factors, such as the physiological stage of the plant and the climate where it grows (heat, photoperiod, humidity), can also affect the yield of an EO [3].

The chemical composition of the *D. winteri* EO has been described from different canelo tissues collected in central and southern Chile. The reports indicate variations in the chemical composition of the oils both qualitatively and quantitatively, highlighting a high content of sesquiterpenes (>50%) [56,57,58,59,60,61]. The CEO that we studied showed a monoterpene (28.08%) and sesquiterpene (56.60%) content similar to oil composition from canelo leaves collected in Quilpué (Valparaiso Region, Chile), which was composed of 30.7% monoterpenes and 60.7% sesquiterpenes [56]. The major compounds of Quilpué oil (*γ*-eudesmol (21.7%), elemol (12.0%), terpinen-4-ol (11.6%), *α*-eudesmol (7.4%) and *β*-eudesmol (7.27%)) are consistent with the major components of CEO evaluated here (elemol 13.5%, *γ*-eudesmol 11.4%, *β*-eudesmol 8.5% and *α*-eudesmol 6.4%). Only terpinen-4-ol (1.9%) was less than in the Quilpué EO and α-pinene (7.9%) was more abundant. On the other hand, Zapata et al. [60] reported a *D. winteri* leaf oil with more diverse main constituents, with *γ*-curcumene + NI (NI = non-identified compound) (11.12%) and then a group of five compounds comprising 6–9%, i.e., limonene + myrcene, limonene + NI, trans-caryophyllene, *α*-pinene, sabinene and 4-terpineol. Additionally, Becerra et al. [58] presented a totally distinct chemical profile of *D. winteri* EO from leaves collected in the Bio-Bio Region, Chile. This oil was constituted only by 6 identified compounds containing one monoterpene, azulene (7.6%), one sesquiterpene, shyobunone (4.2%), one diterpene, kaurene (2.8%) and 3 unsaturated hydrocarbons, cyclohexadiene (9.1%), naphthalene (17.4%) and benzocycloheptene (39.0%) as their principal compound. Consequently, the compositional differences of the *D. winteri* EOs observed in the species may be derived from either an abiotic factor such as climatic, seasonal, geographical or a biotic factor, e.g., genotypic variation [62].

A substantial advantage of using EOs is that they are comprised of a varied and large number of compounds that can act in different action sites [63,64]. Moreover, the insecticide activity of an EO is not always linearly dependent upon the content of its main constituents. In many cases the oil minority fraction possesses a high toxic potency and is thus responsible for the higher final activity or synergistic phenomena enhancing the oil insecticidal activity when its main constituents are mixed [65]. Here, the CEO is characterized by the presence of 6 main compounds and the insecticidal activity in some of them was reported. The sesquiterpene elemol was reported for its toxicity against *Drosophila melanogaster* (Meigen) (Diptera: Drosophilidae) [66] and as a repellent against *Aedes aegypti* (L.) (Diptera: Culicidae) [67,68]. In addition, recent studies have shown that different EOs having elemol in their composition were repellent, fumigant and toxic against *Tribolium castaneum* (Herbst) (Coleoptera: Tenebrionidae) [69], insecticidal against *Drosophila suzukii* (Matsumura) (Diptera: Drosophilidae) [70] and *Sitophilus granarius* (L.) (Coleoptera: Curculionidae) [71] and inhibited the development of *C. maculatus* [72]. The attributes of eudesmol compounds acting as insecticidal agents have not been well studied to date, but there are reports in which these compounds are part of the chemical profile of different EOs with insecticidal action. For example, the *D. winteri* EO caused a mortality of 68% with 64 μL L^−1^ against *Acyrthosiphon pisum* (Harris) (Homoptera: Aphididae) [60] and the *Helichrysum faradifani* Sc. Ell. (Asteraceae) EO was toxic at 85.7 μL L^−1^ on the larvae of *Culex quinquefasciatus* (Say) (Diptera: Culicidae) [73]. Both EOs contained eudesmol and their isomers in their composition. Other sesquiterpenes present in CEO with biologic activity are drimenol and drimenin, which were insecticidal against *S. granarius* [25], while the phenylpropanoid myristicin was larvicidal towards *A. aegypti* [74] and *Spilarctia obliqua* (Walker) (Lepidoptera: Erebidae) [75]. Myristicin has been described for its synergistic activity [76] as occurs with safrole, which is a precursor in the synthesis of the insecticide synergist piperonyl butoxide [77] and acts as a natural antifeedant [78].

The toxicology effect produced by CEO on *A. obtectus* mortality was dose-dependent and probably occurred through inhalation and contact of its compounds, because the adult insects do not feed [34]. This is the first report on the toxicological effect of CEO against bean weevil, *A. obtectus*. After 24 h, an evident toxicological effect of CEO showed a quick mortality of the insects (94%) with the highest dose evaluated (158.3 µL L^−1^). Subsequently, by increasing the exposure time (48 h and 72 h) and decreasing the CEO concentrations (58.3 and 108.3 µL L^−1^), mortality rates of 94% and 100%, were reached. Similarly, Çetin et al. [79] and Gokturk et al. [80] reported high death rates (100%) on *A. obtectus* after 24 h with *Rosmarinus officinalis* (L.) (Lamiaceae) EO. In addition, Gokturk et al. [80] indicated mortality rates > 98% after 72 h exposure with the *Artemisia dracunculus* (L.) (Asteraceae) and *Ocimum basilicum* (L.) (Lamiaceae) EOs against this insect. By contrast, Ayvaz et al. [51] reported 100% mortality with *Origanum onites* (L.) (Lamiaceae) and *Satureja thymbra* (L.) (Lamiaceae) EOs, but with a longer time (144 h) and a higher concentration (195 µL L^−1^) than in our study. Other studies have also reported high doses of EOs to produce a mortality of 99% on *A. obtectus*, 359.2 µL of *Syzygium aromaticum* (L.) (Myrtaceae) EO and 268.0 µL of *Cinnamomum zeylanicum* (L.) (Lauraceae) EO kg^−1^ of stored beans [81]. Interestingly, terpenoids are major constituents of EOs [3]. Papachristos et al. [65] showed fumigant activity of various terpenoids, among them, the monoterpenoids terpinen-4-ol, 1,8-cineole and camphor against *A. obtectus*. Similarly, Regnault-Roger and Hamraoui [82] reported *α*-pinene and terpineol as being toxic and reproduction inhibitors for the bean weevil and they indicated that the most active compounds had an oxygenated structure. The susceptibility of *A. obtectus* to oxygenated terpenoids could be related to an inhibition of acetylcholinesterase [83] or with oxido-reduction reactivity [82]. In our study, the toxicological effect could be due to the high content of oxygenated terpenes (49.5%) identified in the CEO, which could be more active in *A. obtectus*.

On the other hand, the results proved that the repellent activity observed in both sexes of the raspberry weevil is related to inhalation of the volatile compounds released by CEO. Generally, *A. superciliosus* spent more time in the control olfactometer arms compared to the treated arms, which proved the repellent activity of the canelo oil. Similar studies on bioactivities of volatile compounds released from the EOs against pest insects have also been reported. For instance, Tampe et al. [48] reported that *Ruta chalepensis* L. (Rutaceae) EO produced repellency at the same dose that we evaluated (1 µL of pure EO) against *A. superciliosus*. Espinoza et al. [47] showed similar results by evaluating EO from *Pilgerodendron uviferum* (D. Don) Florin (Cupressaceae) against the same insect. Moreover, Tampe et al. [49] also reported repellency from *Achillea millefolium* L. EO on another curculionid, *Aegorhinus nodipennis* (Hope) (Coleoptera: Curculionidae) and Rebolledo et al. [28] described the insecticidal effect of *D. winteri* EO on *A. superciliosus*, with 100% mortality at a concentration of 40% *v/v*. Other studies have reported that *Ruta graveolens* L. (Rutaceae) extracts can produce different effects on *C. capitata*, being able to attract and stimulate oviposition and at the same time act as an insecticide [84]. Generally, plant compounds that act as insect repellents also act as semiochemicals that alter the behavior of an arthropod. Insect repellents work by providing a vapor barrier, deterring the insect from coming into contact with the stimulus [7]. To date, there are no studies reporting on the main compounds of CEO: elemol, *γ*-eudesmol, *β*-eudesmol or *α*-eudesmol by producing any effect on the raspberry weevil. We only found two studies that indicate that *A. superciliosus* perceives the monoterpenes *α*- and *β*-pinene, but they do not individually affect the insect’s behavior [52,85]. In our study, the CEO repellent effect could be produced by any of the main compounds or by the minority fraction of the oil, which has many molecules described with insecticidal action in other insects. This could trigger a synergistic action among them and possibly be responsible for the repellency observed against *A. superciliosus*.

Further studies are required to develop a suitable formulation with this oil. It is necessary to evaluate the individual constituents’ effects and the synergy among them, an action that could improve its efficiency. Finally, knowing the chemical profile of an EO is particularly important in predicting its effect on different insects, how they interact and work in combination with other compounds [86]. However, we cannot generalize from such findings as each substance differs strongly in its activity and varies according to the species.

## 5. Conclusions

This is the first report of the chemical profile of canelo leaves and shoots essential oil obtained from the Araucanía, which enriches the phytochemical knowledge of the species. It was possible to identify 56 compounds from canelo essential oil comprised mainly of elemol, *γ*-eudesmol, *β*-eudesmol and α-eudesmol sesquiterpenes and the *α*- and *β*-pinene hydrocarbonated monoterpenes, showing similarity and variations with the chemical profiles described for *D. winteri*. Moreover, our results indicate that the canelo leaves and shoots essential oil is toxic to *A. obtectus* and produces repellency against *A. superciliosus*. Therefore, *D. winteri*, an endemic species in Chile, is a promising source of phytochemicals for agriculturally important pest control. This work is an advance in the study of essential oils with insecticide properties and contributes to the valorization of endemic species, which may represent an interesting source of biologically active compounds. However, it would be enriching to expand knowledge of interactions among several essential oils from endemic plants in Chile in order to lead to synergistic, additive or antagonistic effects against pests posing a risk to commercial interests.

## Figures and Tables

**Table 1:** Chemical composition of the *Drimys winteri* leaves and shoots essential oil.

Peak	RT	RI	Compound	%	Identification
1	8.17	922	*α*-thujene	0.08	RI, MS
2	8.36	929	***α*** **-pinene**	7.92	RI, MS
3	8.66	940	camphene	0.24	RI, MS
4	9.37	965	sabinene	3.20	RI, MS
5	9.45	968	***β*** **-pinene**	5.17	RI, MS
6	9.91	983	*β*-myrcene	0.76	RI, MS
7	10.24	993	*α*-phellandrene	0.10	RI, MS
8	10.60	1005	*α*-terpinene	0.56	RI, MS
9	10.89	1016	eucalyptol	1.41	RI, MS
10	10.98	1019	limonene	3.08	RI, MS
11	11.25	1029	*β*-*trans*-ocimene	1.16	RI, MS
12	11.83	1049	*γ*-terpinene	0.97	RI, MS
13	12.71	1078	terpinolene	0.26	RI, MS
14	13.70	1111	3-octyl acetate	0.10	RI, MS
15	14.01	1122	*allo*-ocimene	0.06	RI, MS
16	14.78	1150	borneol	0.05	RI, MS
17	15.15	1162	(-)-terpinen-4-ol	1.90	RI, MS
18	15.51	1174	*α*-terpineol	1.13	RI, MS
19	18.10	1267	safrol	0.22	RI, MS
20	20.16	1344	*α*-cubebene	0.88	RI, MS
21	20.82	1370	copaene	0.18	RI, MS
22	21.18	1383	*β*-elemene	1.02	RI, MS
23	21.85	1409	caryophyllene	0.82	RI, MS
24	22.10	1420	*β*-cubebene	0.04	RI, MS
25	22.36	1431	aromadendrene	0.03	RI, MS
26	22.66	1443	*α*-caryophyllene	0.23	RI, MS
27	23.16	1463	(+)-*epi*-bicyclosesquiphellandrene	0.69	RI, MS
28	23.32	1470	germacrene d	3.18	RI, MS
29	23.54	1478	*γ*-muurolene	0.43	RI, MS
30	23.69	1484	*γ*-elemene	1.83	RI, MS
31	23.83	1490	myristicin	4.66	RI, MS
32	24.08	1500	*γ*-cadinene	0.39	RI, MS
33	24.17	1504	(-)-calamenene	0.49	RI, MS
34	24.30	1509	(-)-*β*-cadinene	1.14	RI, MS
35	24.52	1519	(+)-*δ*-cadinene	0.15	RI, MS
36	24.75	1529	hedycaryol	1.93	RI, MS
37	24.90	1536	**elemol**	13.54	RI, MS
38	25.19	1549	*e*-nerolidol	0.28	RI, MS
39	25.44	1559	spathulenol	0.22	RI, MS
40	25.67	1569	globulol	0.45	RI, MS
41	25.83	1576	ledol	0.31	RI, MS
42	26.77	1617	***γ*** **-eudesmol**	11.42	RI, MS
43	27.10	1632	***β*** **-eudesmol**	8.49	RI, MS
44	27.23	1638	***α*** **-eudesmol**	6.39	RI, MS
45	27.45	1648	bulnesol	0.50	RI, MS
46	28.00	1673	eudesm-7(11)-en-4-ol	0.14	RI, MS
47	29.42	1740	drimenol	1.41	RI, MS
48	32.56	1893	sclaren	0.04	MS
49	32.72	1901	drimenin	0.01	MS
50	32.99	1916	rimuen	0.46	RI, MS
51	34.16	1977	ethyl palmitate	0.04	RI, MS
52	34.94	2019	kaur-16-ene	0.65	RI, MS
53	37.08	2138	ethyl linoleate	0.06	RI, MS
54	37.23	2147	ethyl oleate	0.24	RI, MS
55	37.78	2179	ethyl stearate	0.01	RI, MS
56	44.51	2601	hexacosane	1.11	RI, MS
-	-	-	monoterpenes	28.08	-
-	-	-	sesquiterpenes	56.60	-
-	-	-	diterpenes	1.15	-
-	-	-	phenylpropanoids	4.88	-
-	-	-	others	1.57	-

RT—retention time (min); RI—Kovats retention index; %—considering detected compounds; MS—mass spectra. Compounds written in bold correspond to the most abundant compounds detected in canelo leaves and shoots essential oil (CEO).

**Table 2:** Toxicological activity of *D. winteri* essential oil against *A. obtectus* adults.

Time (h)	LC_50_ (95% FCI)	LC_90_ (95% FCI)	χ^2^	p	Slope (±SE)
24	60.1 (49.87–70.49)	163.05 (139.20–196.09)	9.04	<0.001	−3.85 ± 0.42
48	14.8 (11.24–19.05)	40.18 (31.14–53.42)	8.09	<0.001	−1.63 ± 0.20
72	11.15 (8.91–13.8)	30.26 (24.04–39.75)	7.15	<0.001	−1.31 ± 0.18

Dose causing 50% and 90% mortality; unit LC_50_ and LC_90_ = µL L^−1^, applied for 24, 48 and 72 h. χ^2^ = chi-squared value. FCI—fiducial confidence interval.
